# Targeting molecular networks for drug research

**DOI:** 10.3389/fgene.2014.00160

**Published:** 2014-06-04

**Authors:** José P. Pinto, Rui S. R. Machado, Joana M. Xavier, Matthias E. Futschik

**Affiliations:** ^1^SysBioLab, Centre for Molecular and Structural Biomedicine, Universidade do AlgarveFaro, Portugal; ^2^Centre of Marine Sciences, Universidade do AlgarveFaro, Portugal

**Keywords:** networks, molecular interactions, drugs, diseases, stem cells

## Abstract

The study of molecular networks has recently moved into the limelight of biomedical research. While it has certainly provided us with plenty of new insights into cellular mechanisms, the challenge now is how to modify or even restructure these networks. This is especially true for human diseases, which can be regarded as manifestations of distorted states of molecular networks. Of the possible interventions for altering networks, the use of drugs is presently the most feasible. In this mini-review, we present and discuss some exemplary approaches of how analysis of molecular interaction networks can contribute to pharmacology (e.g., by identifying new drug targets or prediction of drug side effects), as well as list pointers to relevant resources and software to guide future research. We also outline recent progress in the use of drugs for *in vitro* reprogramming of cells, which constitutes an example* par excellence* for altering molecular interaction networks with drugs.

## INTRODUCTION

Over the last decade, we have witnessed impressive technological advances in the field of molecular biology. Many of them have brought us an incredible wealth of molecular data. Initially, it was hoped that large data-driven projects such as the Human Genome Project would readily pave the way for the development of new effective therapies in biomedicine. Unfortunately, the translation of these molecular data into biomedical breakthroughs has been dauntingly slow. Why is this so?

One reason for this “bottleneck” is that biological processes are highly interconnected, so their manipulation is a formidable challenge. In addition, major human diseases, such as cancer, type II diabetes, and hypertension, are genetically complex. Hence, a direct correspondence between causative genotype and disease phenotype, as observed in Mendelian disorders, is frequently obscure. Instead, these diseases are multi-factorial and seem to result from interplay between multiple genes and environmental factors, each having a relatively small effect, with few (if any) being prerequisites for the disease to occur (Manolio, 2010). This view is supported by several other lines of investigations that underline how important it is to regard causative genes not as isolated entities, but as integral parts of molecular networks or pathways (Badano and Katsanis, 2002; Oti and Brunner, 2007).

## MOLECULAR NETWORKS: DATA AND ANALYSIS

In recognition of the importance of molecular networks, researchers from different fields have begun to study them intensely through computational and experimental means. Their underlying premise has been that changes to cellular networks determine many phenotypic variations, and that such changes can be provoked, not only by alterations to a gene product’s abundance, but also through perturbations of its interactions.

The intensified interest in molecular networks has resulted in systematic gathering of interaction data for biomolecules, as well as the development of computational approaches for the analysis of biological networks. Nowadays, a large number of publicly accessible databases contain various types of molecular interaction data^1^. Networks derived from these resources frequently contain only a specific type of molecular interaction such a protein–protein or protein–DNA interactions. Based on the type of included interaction, we distinguish between different types of interaction networks. Currently, the major types are protein–protein interaction (PPI), gene regulatory and metabolic networks. These networks are often visually represented as simple graphs, with nodes or vertices denoting molecules, and links or edges denoting interactions between them. While such drastic simplification neglects many characteristics of individual components, it facilitates the analysis and modeling of large cellular networks. Furthermore, we can profit from the rich repertoire of mathematical tools and concepts already developed in graph theory.

**FIGURE 1:**
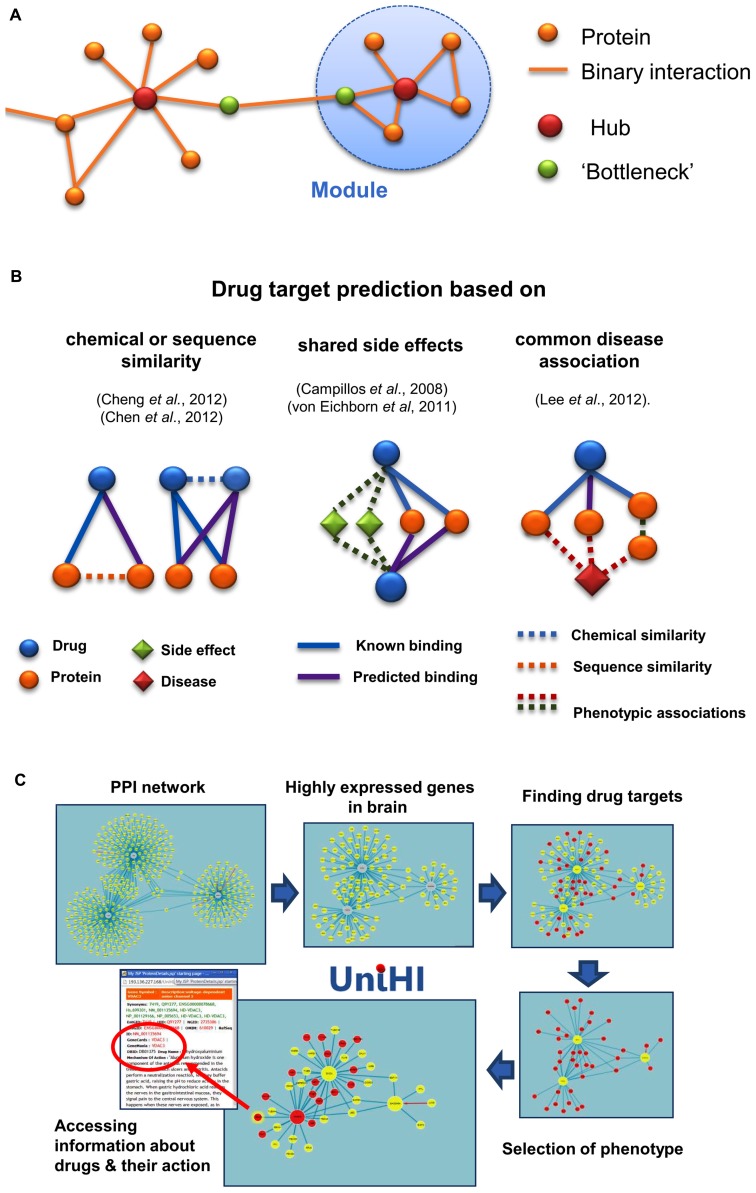
**(A)** Illustration of basic concepts in the analysis of molecular networks. Hubs are defined by their large number of interactions, whereas “bottleneck” proteins link densely connected sub-networks or modules. Both types of nodes provide prominent targets for interventions, aimed at changing the network structure and integrity. **(B)** Approaches for network-based drug targeting and repositioning. Different types of heterogeneous bipartite or tripartite networks have been used in the literature to identify new targets for drugs. **(C)** Network-oriented pharmacology in the UniHI environment. After querying for molecular interactions for central proteins, UniHI derives tissue and phenotype-specific networks, which can be scrutinized for known drug targets. In the example shown, an interaction network with *GADD45A*, *SNCA*, *PARK2* as central proteins was retrieved and filtered using gene expression data from the brain. Additional filtering steps, using drug–target data and phenotypic information (“nervous system phenotype”) from knock-out mice, generated a compact network of drug targets with potential relevance for neurological disorders. Information regarding the drugs and their mode of action can be interactively accessed within the displayed network.

The most basic characteristic of a node in a graph is its *degree*, i.e., the number of edges attached to it. In many biological networks, the majority of nodes have a low degree, and only a few nodes have a high degree. These highly connected nodes are known as hubs, and are important for the integrity of the network (Albert, 2005). Another important concept in graph theory is *modularity*. A module is commonly regarded as a set of nodes that are more densely connected with each other than with other nodes in the network (Pinto, 2012). These two concepts are illustrated for biological networks in **Figure 1A**. Modularity has also been suggested to contribute to *robustness* of molecular systems (Hartwell et al., 1999). In fact, robustness of molecular processes seems to result directly from the structure of the underlying networks. Besides redundant genetic components, compensatory network structures such as alternative metabolic or signaling pathways can buffer the failure of single parts (Wagner, 2005). This feature of networks is a crucial aspect to be considered, when we want to design effective interventions in their functioning.

Prime examples of popular and freely available software for network analysis are R/Bioconductor^2^ or Cytoscape^3^. While these are powerful and versatile tools, their use requires expertise in both data handling and processing. Alternatives are given by several on-line resources, which provide integrated and annotated data together with applications for analysis and visualization. For instance, our Unified Human Interactome (UniHI)^4^ database stores a large number of molecular interactions for the human genome, together with other types of information, and includes tools for the interactive analysis of retrieved interaction networks (Chaurasia et al., 2007; Kalathur et al., 2014). Especially for researchers less acquainted with network analysis, such integrative platforms offer convenient gateways to a wealth of interaction data.

## DRUGS AND THEIR TARGETS

Pharmaceutical drugs are a common means to modify the activity of biomolecules, making them prime candidates for altering activity and structure of molecular networks as well. The targets of drugs can be proteins, peptides or nucleic acids, whose activities can be modulated. Drugs can be sub-divided into at least three different classes: (i) chemical compounds with low molecular weight (typically referred to as small molecules) that target enzymes, receptors, transcription factors or ion channels; (ii) biologics (such as antibodies or recombinant proteins) that target extracellular proteins and transmembrane receptor; and (iii) nucleic acids that target messenger RNA by interference (Gashaw et al., 2011). Notably, small molecules are still by far the most common type of drugs, and are frequently associated with low costs and easy (i.e., oral) delivery. However, the number of proteins, which can be targeted by small molecules, appears to be fairly limited (Overington et al., 2006).

Ideally, drug targets should have: (i) a proven role in the pathophysiology of a disease; (ii) little impact on physiological (health) conditions when modulated; and (iii) a favorable prediction for potential side effects (Gashaw et al., 2011). To fulfill the later criterion, highly selective targeting is generally considered to be a desirable trait. To target multiple proteins, as is frequently required for treatment of complex diseases, it is therefore necessary to combine multiple drugs. Especially for cancer, combinatorial drug therapy has become a standard practice, minimizing the risk of drug resistance. However, kinase inhibitors, which target multiple pathways simultaneously, have shown efficacy in the treatment of different cancers (Al-Lazikani et al., 2012). Thus, it has been argued that multiple-target drugs might be a more favorable option, since detrimental drug–drug interactions can be avoided, and optimal dosage can be more easily determined (Hopkins, 2008).

## NETWORK-BASED APPROACHES FOR DRUG RESEARCH

### IDENTIFICATION OF DRUG TARGETS

The identification of drug targets is a crucial, but laborious task in biomedical research. Nowadays, *in silico* methods can assist greatly. Conventional *in silico* methods for drug target prediction are typically receptor- or ligand-based models. Whereas receptor-based methods start with a known structure of the target, and employ docking to assess drug binding (Luo et al., 2011); ligand-based methods involve the comparison of drugs with known ligands of the target protein. A successful example of the latter method on a genomic scale is the study by Keiser et al. (2009), in which a large number of new potential targets for existing drugs were found based on chemical similarity with known ligands.

More recently, network-based methods have complemented the computational toolbox for drug target identification. They are especially helpful, if the three-dimensional structure of the target is unknown. Network-based methods are motivated by the observation that the general biological importance of a protein is at least partially linked to its location in relevant PPI networks. For instance, essential genes tend to correspond to hubs or central nodes in many PPI networks; although, in practice, such conclusions might be compromised by prevalent inspection biases (Futschik et al., 2007; Barabási et al., 2011). Consequently, drugs should target central nodes, when a lethal effect is intended, as it is the case, for example, in the treatment of cancer cells or pathogens (**Figure 1A**). In contrast, if a molecular process needs be adjusted, it might be preferable to target neighbors of central nodes (Csermely et al., 2013). This approach is consistent with observations that targets of approved drugs tend to have more connections on average than most proteins, but fewer connections than for those proteins that correspond to essential genes (Yildirim et al., 2007).

In addition to degree as a basic centrality measure, other more sophisticated local metrics, including bridging centrality and graphlet degree, have been proposed for the identification of drug targets in PPI networks (Hwang et al., 2008; Milenkoviæ et al., 2011). Alternatively, global network-based analyses can be used to provide cues for follow-up investigations. For example, a systematic review of major signaling pathways led to the conclusion that proteins involved in cross-talk between pathways, represent promising targets for drug (Korcsmáros et al., 2010).

While the study of the topology of PPI networks provides a valuable, general indication about the likelihood of finding drug targets; more specific predictions can be determined by evaluating local heterogeneous networks (**Figure 1B**). One of the first steps in this direction was taken in the work of Yamanishi et al. (2008), who transformed a bipartite network (in which two types of nodes form a network) of drugs and their known targets into a high dimensional composite “pharmacological feature space”, where interacting drugs and targets were close to each other. New chemicals or targets could be mapped into this feature space, and drug–target interactions were predicted based on their spatial proximity. A simpler approach, based on diffusion of scores within the local bipartite network neighborhood, has recently been proposed. This approach outperformed predictions based on interference using either chemical similarity of drugs, or sequence similarity of targets (Cheng et al., 2012). Although several of its predicted new targets of known drugs were successfully validated, a drawback of this simpler method is that it cannot be applied to novel drugs. This limitation can be overcome through integration of the drug–target network with drug–drug (based on chemical similarity) and target–target (based on sequence similarity) networks. In the study by Cheng et al. (2012), random walks on these integrated heterogeneous networks were simulated to connect drugs with potential targets. Using drug–drug connections, new drugs, for which no target is yet known, can be linked to proteins via drugs that have known targets.

Furthermore, the use of expression responses appears to assist in the process of drug target identification. Starting with a network of functional associations between proteins, Laenen et al. (2013) evaluated whether differential gene expression upon drug treatment can pinpoint the protein targeted by a drug. Strikingly, while the expression changes of the target itself was only moderately informative, integration of differential expression observed in the target’s network neighborhood resulted in a drastic increase in prediction accuracy. However, it remains to be assessed, whether it is generally the case that expression of genes functionally related to a target is altered by its corresponding drug.

### REPOSITIONING OF DRUGS

Closely related to drug target identification is the task of drug repositioning, i.e., finding new therapeutic uses for existing drugs (Tobinick, 2009). Since drug repositioning is based on known drugs, it provides an attractive shortcut to the lengthy development of new drugs. While the above mentioned approaches for drug target identification also can be applied to drug repositioning, several methods and software have been exclusively developed for this task. For instance, Mathur and Dinakarpandian (2011) proposed new possible disease–drug relationships through the analysis of affected biological processes. After identifying processes defined in Gene Ontology that were enriched by genes associated with a particular disease, drugs were linked to these processes, if they targeted central proteins of the PPI network representing these processes. Through comparing predicted disease–drug relationships with ones that had been reported in clinical trials, they found a statistically significant overlap. A similar, but more direct approach has been implemented in the PharmDB database, which integrates binary linkages between drug, proteins, and diseases (Lee et al., 2012). New targets of existing drugs are inferred using a method called Shared Neighborhood Scoring, which evaluates weighted connections between drug and disease nodes via their associated proteins in a tripartite network composite. An alternative software tool, which combines structural models with analysis of interaction profiles, is DRAR-CPI (Luo et al., 2011). This web-server compares the binding behavior of a candidate drug with a set of pre-determined drug–target interactions using a docking approach. Similar interaction profiles can indicate shared targets and common clinical application. The number of included reference targets for docking, however, is limited.

It is important to note, that the use of networks as computational tools is not necessary constrained to the representation of actual molecular interactions, but can be used to represent any kind of defined similarities or association between distinct entities. For instance, Iorio et al. (2010) derived a drug–drug network, where links between drugs indicated similar expression changes upon treatment; they exploited it both for drug target prediction, as well as repositioning.

### ANALYSIS OF SIDE EFFECTS

Physiological side effects can be caused by binding of drugs to proteins (“off-targets”), in addition to their intended targets. As side effects are crucial factors in therapeutic applications, their accurate prediction is of eminent importance to avoid failure in drug trials. Notably, systematic recording of side effects represents a broad phenotying on the level of the human organism, providing valuable holistic information on the action of drugs. A unique resource, with this objective, is the SIDER database, which accumulates reported side effects for almost 1000 marketed drugs (Kuhn et al., 2010). Using this database, Mizutani et al. (2012) correlated a drug’s side effects with the proteins it binds to. For this, side effects and bound proteins were represented as binary profiles and statistically associated using a modified version of canonical correlation analysis. The obtained correlation was used subsequently for the prediction of side effects, by evaluating the proteins that the drug binds to. Remarkably, it is equally possible to predict a drug’s target based on its side effects. This relationship was originally explored by Campillos et al. (2008); they identified new targets of known drugs based on the similarity of their side effects with those of other drugs. There is now a database, which has implemented this approach, called PROMISCUOUS (von Eichborn et al., 2011). It enables the interactive exploration of an integrated network of drug, protein, and side effect nodes, and can be used to gain new insight into the drug’s mode of action. Finally, side effects can also be indicative for drug–drug interactions, which are frequently of clinical relevance. It was recently shown that two drugs tend to interact, if their targets are in close proximity in a PPI network, or if they have similar side effects (Huang et al., 2013). Moreover, combining information on physical interaction of drug targets and recorded side effects improves the prediction accuracy for drug–drug interactions.

In **Table 1**, we provide a selection of publicly available databases and computational resources, which may be useful for the reader to initiate their own investigations in the field of network-based pharmacology.

**Table 1:** Publically available resource for network-based drug targeting and repositioning.

Resource	URL	Description	Reference
DRAR-CPI	http://cpi.bio-x.cn/drar/	Web server that derives and compares the interaction profile of a inputted drug with those of a library of drugs	Luo et al. (2011)
DrugBank	http://www.drugbank.ca/	Database containing detailed information for approved or experimental drugs and their targets	Knox et al. (2011)
DvD	http://www.ebi.ac.uk/saezrodriguez/DVD/	Add-on software packages for R and Cytoscape for drug repurposing using gene expression data	Pacini et al. (2013)
Mantra	http://mantra.tigem.it/	Computational on-line tool for analyzing the mode of action of a drug using its induced gene expression	Iorio et al. (2010)
PROMISCUOUS	http://bioinformatics.charite.de/promiscuous	Database for drug repositioning based on integrated PPI, drug–protein interactions, and side effects	von Eichborn et al. (2011)
SIDER	http://sideeffects.embl.de	Database containing side effects of marketed drugs	Kuhn et al. (2010)
Stitch	http://stitch.embl.de/	Database accumulating a large number of interactions between chemicals and proteins for various organisms	Kuhn et al. (2012)
UniHI	http://www.unihi.org	Web-based platform integrating human molecular interactions, gene expression, phenotypes, and drug target information (**Figure 1C**)	Kalathur et al. (2014)

## NEW HORIZONS: *IN VITRO* REPROGRAMMING OF CELLS USING SMALL MOLECULES

In the network-based approaches described above, drugs mainly act within small sub-networks in order to “fix” or interfere with particular processes. This contrasts with their recent use in stem cell biology, where small molecules have been used to re-wire entire cellular networks. Their main object in this context is to convert (or reprogram) somatic cells, specific to an individual, into stem cells. These cells may eventually provide a personalized supply of tissue to replenish cells lost in degenerative diseases. Pioneering work led by Yamanaka showed that such conversion is possible through forced expression of merely four transcription factors using viral vectors (Takahashi and Yamanaka, 2006). The original combination of transcription factors used by Yamanaka comprises Octamer-binding transcription factor 4 (Oct4), Sex-determining region Y-box 2 (Sox2), Kruppel-like factor 4 (Klf4), and v-myc avian myelocytomatosis viral oncogene homolog (c-Myc). However, this approach suffers from low efficiency. Furthermore, the viral integration of exogenous transcription factors, in particular of oncogenes, such as Klf4 and c-Myc, is unlikely to offer a viable therapeutic option. Thus, efforts have been made by various groups to find small molecules that can boost reprogramming efficiency, as well as replace virally transduced transcription factors.

Two main classes of small molecules have been identified so far: (i) molecules that facilitate chromatin remodeling by inhibition of, e.g., histone deacetylase, and thereby increase the plasticity of cells (Huangfu et al., 2008); and (ii) molecules that block signaling events that induce differentiation. Examples of the latter class are inhibitors of extracellular signal-regulated kinases (ERKs) and glycogen synthase kinase 3 (GSK3; Silva et al., 2008). By combining these two classes of small molecules, it is even possible to replace all four transcription factors (Hou et al., 2013). A remaining challenge, however, is to determine the underlying molecular processes of chemically induced pluripotency. So far, only rudimentary models, which lack mechanistic details, have been proposed for the activation of key transcription factors by the applied molecules (Hou et al., 2013). Here computational methods for “reverse engineering” of gene regulatory networks can be very helpful. These methods aim to infer regulatory interactions from observed gene expression patterns and comprise a diverse set of statistical approaches such as regression, analysis of correlation or mutational information or Bayesian networks (Marbach et al., 2012). Usually, their application requires a large set of genome-wide expression measurements and might not scale up very well to the complexity of regulatory networks in higher eukaryotes. Nevertheless, a recent study identified successfully a novel regulator of stem cell differentiation through reverse engineering of gene regulatory networks from microarray expression data (De Cegli et al., 2013). We anticipate that such approaches as well as systems biology in general will help to establish a rational basis for creating chemically induced pluripotency.

## PERSPECTIVES

Our review highlights several applications of molecular networks, in which they act as versatile interfaces between phenotypes and drugs. While these applications demonstrate the utility of network-based analyses, several major challenges still exist. Firstly, the quality and coverage of interaction data need to be improved and consolidated. Many interaction data sets suffer from both detection and selection biases, which limit their use (Futschik et al., 2007). Published drug target data also appear to be compromised by their low reproducibility (Prinz et al., 2011). Secondly, condition-specific networks need to be constructed, reflecting the dynamics of molecular processes, in contrast to the static nature of current models. In this way, it will be possible to study the effects of external and internal stimuli on network structure and function. Finally, the vast majority of available drugs target network nodes, disrupting the general activity of a specific biomolecule. Only a small number of drugs are directed towards specific interactions (Wells and McClendon, 2007). Such “link-directed” drugs, however, can provide a more precise means to modulate molecular networks.

In summary, network-based analyses offer new ways of studying targets and effects of drugs. Although challenges lie ahead, network models promise to be powerful and versatile tools in our quest to better understand and control molecular systems in health and disease.

## Conflict of Interest Statement

The authors declare that the research was conducted in the absence of any commercial or financial relationships that could be construed as a potential conflict of interest.

## Footnotes

1 http://www.pathguide.org

2 http://www.bioconductor.org

3 http://www.cytoscape.org

4 http://www.unihi.org
